# Controlled Decompression Attenuates Compressive Injury following Traumatic Brain Injury via TREK-1-Mediated Inhibition of Necroptosis and Neuroinflammation

**DOI:** 10.1155/2021/4280951

**Published:** 2021-11-08

**Authors:** Tao Chen, Xiao Qian, Jie Zhu, Li-Kun Yang, Yu-Hai Wang

**Affiliations:** Department of Neurosurgery, The 904th Hospital of PLA, Medical School of Anhui Medical University, Wuxi, Jiangsu 214044, China

## Abstract

Decompressive craniectomy is an effective strategy to reduce intracranial hypertension after traumatic brain injury (TBI), but it is related to many postoperative complications, such as delayed intracranial hematoma and diffuse brain swelling. Our previous studies have demonstrated that controlled decompression (CDC) surgery attenuates brain injury and reduces the rate of complications after TBI. Here, we investigated the potential molecular mechanisms of CDC in experimental models. The in vitro experiments were performed in a traumatic neuronal injury (TNI) model following compression treatment in primary cultured cortical neurons. We found that compression aggravates TNI-induced neuronal injury, which was significantly attenuated by CDC for 2 h or 3 h. The results of immunocytochemistry showed that CDC reduced neuronal necroptosis and activation of RIP3 induced by TNI and compression, with no effect on RIP1 activity. These protective effects were associated with decreased levels of inflammatory cytokines and preserved intracellular Ca^2+^ homeostasis. In addition, the expression of the two-pore domain K^+^ channel TREK-1 and its activity was increased by compression and prolonged by CDC. Treatment with the TREK-1 blockers, spadin or SID1900, could partially prevent the effects of CDC on intracellular Ca^2+^ metabolism, necroptosis, and neuronal injury following TNI and compression. Using a traumatic intracranial hypertension model in rats, we found that CDC for 20 min or 30 min was effective in alleviating brain edema and locomotor impairment in vivo. CDC significantly inhibited neuronal necroptosis and neuroinflammation and increased TREK-1 activation, and the CDC-induced protection in vivo was attenuated by spadin and SID1900. In summary, CDC is effective in alleviating compressive neuronal injury both in vitro and in vivo, which is associated with the TREK-1-mediated attenuation of intracellular Ca^2+^ overload, neuronal necroptosis, and neuroinflammation.

## 1. Introduction

Traumatic brain injury (TBI) has been considered as one of the most complex human diseases because of the complexity of brain damage mechanisms and poor prognosis. For patients with uncontrollable high intracranial pressure, decompressive craniectomy is an effective strategy to reduce traumatic intracranial hypertension and improve prognosis [1]. However, the standard procedure of rapid decompression is associated with many postoperative complications, such as delayed intracranial hematoma and diffuse brain swelling [2, 3]. Our previous studies have demonstrated that the controlled decompression (CDC) surgery attenuates brain injury and reduces the rates of complications after TBI [4–6]. However, the underlying molecular mechanism has not been determined.

In the central nervous system (CNS), a variety of cellular physiological processes, including neurotransmitter release, neuronal excitability, and plasticity, are regulated by ion channels, especially Ca^2+^ and K^+^ ions [7]. The tandem of pore domain in weak inwardly rectifying K^+^ channel- (TWIK-) related K^+^ channels (TREK) belongs to the recently discovered two-pore domain K^+^ (K_2P_) channels, which include 15 members grouped in six subfamilies and are responsible for maintaining the neuronal resting membrane potential [8]. TREK-1, also known as KCNK2 or K_2P_2.1, is highly expressed in the lung and brain, from the prefrontal cortex, hippocampus, and midbrain to the spinal cord [9]. Since its cloning two decades ago, the physiological importance of TREK-1 is constantly being discovered. TREK-1 is located at both presynaptic and postsynaptic components, which are its key roles in maintaining the resting membrane potential and neurotransmitter release. Its activation is regulated by various physical and chemical stimuli, such as mechanical stretch, Gq-coupled group I mGluRs, and Gs-coupled 5-HR4 receptor serotonin [10]. Increasing evidence has been demonstrated that dysfunction of TREK-1 channels is involved in multiple neurological pathologies, including depression, pain, epilepsy, and ischemia [11]. In TREK-1 gene knockout mice, TREK-1 null was found to increase neuronal excitability and enhance excitatory and inhibitory postsynaptic currents, thereby impairing cognitive function [12]. In addition, TREK-1 was found to be upregulated by ischemia in astrocytes to enhance glutamate clearance and block neuronal death [13]. However, the role of TREK-1 in traumatic intracranial hypertension conditions has not been fully determined.

In the current study, we investigated the effect of CDC on compressive injury following neuronal trauma in an in vitro model using primary cultured cortical neurons and also in an in vivo traumatic intracranial hypertension model in rats. Considering the effect of mechanical force on TREK-1 activation, we assessed the potential role of TREK-1 in CDC-induced protection.

## 2. Materials and Methods

### 2.1. Primary Culture of Cortical Neurons

Because neurons in the cerebral cortex are most vulnerable under traumatic intracranial hypertension, primary cultured cortical neurons were obtained from SD rats to establish the experimental models as previously described [14]. All the animal research procedures were approved by the Anhui Medical University Committee of Animal Research.

### 2.2. In Vitro Model and Treatments

The TNI model was performed according to our previously published method [15]. In brief, traumatic injury was performed on cultured neurons by using a rotating scribe injury device, which consisted of a rotating cylinder with ten holes, steel needles, and a permanent magnet. The cylinder holes are distributed at the same interval from the center, and these holes allowed the ten steel needles to freely cross through. A magnet is placed under the culture dish, which ensured that the steel needles could cling to the cell layer as the cylinder rotated. After one turn of this device, ten concentric circular scratches were produced in the neuronal layer with equal distances (1.5 mm) between the scratches. After TNI, neurons were exposed to continuous high pressure of 0.5 MPa according to our previous published paper [16]. Briefly, a custom-made compression apparatus in vitro was applied to expose neurons to continuous high pressure for 3 h. The TREK-1 blockers, spadin and SID1900, were obtained from the Mycell Biotech Company (Shanghai, China) and diluted in neurobasal medium to achieve the final concentration of 1 *μ*M or 30 *μ*M, respectively.

### 2.3. Hematoxylin-Eosin (H&E) Staining

At 12 h after TNI and compression treatment, cortical neurons were washed with phosphate buffer saline (PBS) and fixed with 4% paraformaldehyde for 30 min. Neurons were deparaffinized with xylene, stained by H&E, and then assessed by a pathologist for neuronal loss under an Olympus U-DO3 light microscope (Tokyo, Japan).

### 2.4. LDH Release

The neurotoxicity in cortical neurons was determined by measuring LDH release at 12 h after TNI and compression using a kit according to the manufacturer's protocol (Jiancheng Bioengineering Institute, Nanjing, Jiangsu, China).

### 2.5. Cell Viability Assay

Cell viability of cortical neurons was determined by calcein AM assay at 12 h after TNI and compression using a kit according to the manufacturer's protocol (Enzo Life Sciences, Farmingdale, NY, USA).

### 2.6. Immunocytochemistry

For immunocytochemistry, the neurons were cultured in poly-D-lysine-coated coverslips and treated with TNI, compression, or CDC. After being fixed with 4% paraformaldehyde, permeabilized with 0.1% Triton X-100, and washed with PBS for three times, neurons were blocked by 5% bovine serum albumin. Incubation with the RIP1 (1 : 100, #3493, Cell Signaling), RIP3 (1 : 200, ab62344, Abcam), and TREK-1 (1 : 50, sc-398449, Santa Cruz) primary antibodies was performed at 4°C overnight. After being washed by PBS with Tween-20 (PBST) for three times, the samples were incubated with the secondary antibody at 37°C for 1 h. Then, incubation with 4′,6-diamidino-2-phenylindole (DAPI) was performed to stain the nuclei, and the images were obtained using a Leica SP5 II confocal microscope.

### 2.7. Enzyme-Linked Immunosorbent Assay (ELISA)

The levels of inflammatory cytokines, including TNF-*α*, IL-1*β*, IL-6, INF-*γ*, and IL-18, were measured by ELISA kits following the manufacturer's protocols (Anoric-Bio, Tianjin, China).

### 2.8. Ca^2+^ Imaging

Ca^2+^ imaging was performed using the Ca^2+^ indicator Fura-2 AM to measure the intracellular Ca^2+^ concentrations [17]. The neurons cultured in coverslips were loaded with 5 *μ*M Fura-2 AM in HBSS solution for 30 min and equilibrated lucifugally for 30 min. Cells were excited at 345 and 385 nm using a confocal laser scanning microscope, and the emission fluorescence at 510 nm was recorded. The fluorescence values were then plotted against time and shown as *F*/*F*_0_.

### 2.9. Patch Clamp Electrophysiology

The single TREK-1 channel currents were recorded in cortical neurons using the inside-out patch clamp configuration, and the bath solution contained the following: 130 mM KCl, 10x HEPES, 1 mM MgCl_2_, 5 mM EGTA, and 10 *μ*M Ca^2+^. The experiments were performed at membrane voltages of -40 mV. TREK-1 currents were filtered at 1 kHz and digitized at 5 kHz. Analysis was performed using Clampfit 9.2 (MDS Analytical Technologies).

### 2.10. Traumatic Intracranial Hypertension Model

**Figure 1:**
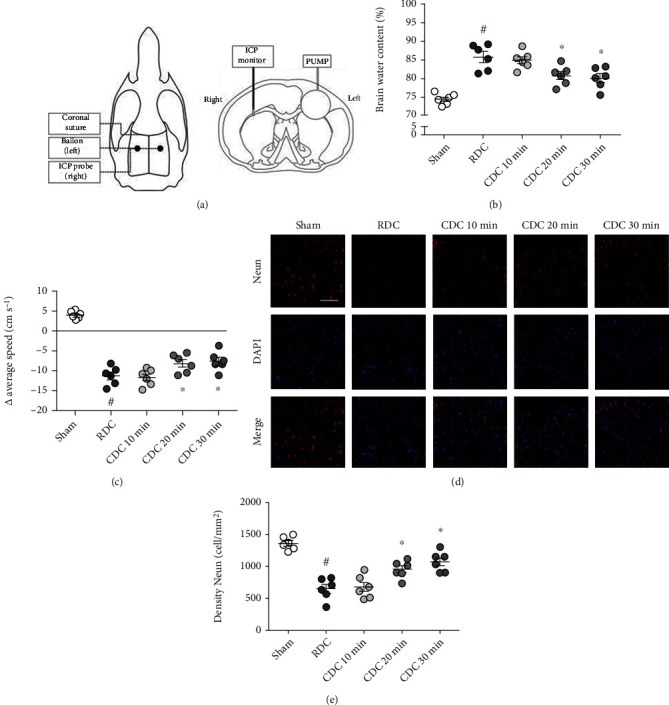
CDC attenuates brain damage after traumatic intracranial hypertension. (a) The horizontal and coronal schematic diagrams of the traumatic intracranial hypertension model. (b) Brain water content assay shows that CDC for 20 min or 30 min reduced brain edema compared to RDC. (c) The neurological assay shows that CDC for 20 min or 30 min attenuates locomotor impairment compared to RDC. (d, e) Typical pictures of NeuN staining (d) and quantification (e) show that CDC for 20 min or 30 min decreases neuronal loss compared to RDC. The data was represented as means ± SEM. ^#^*p* < 0.05 vs. sham group and ^∗^*p* < 0.05 vs. RDC group.

Traumatic intracranial hypertension was induced using a balloon compression method in SD rats. Briefly, anesthesia was induced via 5% isoflurane (RWD, Guangdong, China) with a 1 : 1 N_2_O/O_2_ mixture and was then maintained with a 2.5% isoflurane with 1 : 1 N_2_O/O_2_ mixture by a face mask. The rats were placed on a fixing frame in the prone position. 2% lidocaine was injected subcutaneously near the midline of the skull to minimize the influence of skin incision on blood pressure. The fur around the midline of the skull was cleared with an electronic clipper and the skin of the rat was disinfected by using 75% alcohol. We incised the scalp along the midline for 2.5 cm, separated the subcutaneous tissue, and striped the periosteum to expose the anterior- and posterior-sagittal suture adequately. We made two holes using a dental drill (JSDA, JD700, China) bilaterally in the position of 0.5 cm by the side of the midline and 0.6 cm behind the anterior-sagittal suture and carefully removed the skull fragment to expose the cerebral dura mater without inflicting additional damage. The balloon system is composed of an embolization balloon (Balt 2, Montmorency, France) mounted on an intervention catheter (MAGIC, Montmorency, France). The balloon was inserted into the left hole carefully towards the frontotemporal side, and the dura mater was kept intact during the insertion so that the balloon would only lead to epidural compression. The catheter was connected to a pressure pump (Merit Basix Touch Inflation Device, IN4130, USA) to control the amount of injection fluid accurately. The ICP transducer was inserted into the brain at the depth of around 0.5 cm and was connected to an ICP monitoring device (Codman, Johnson and Johnson Medical, 82-6635, USA). After the insertions of the ICP probe and balloon, the bone holes were sealed using dental cement (Dentsply, Jeltrate Alginate Impression Material, USA) (Figure 1(a)). When the balloon was inflated by the pressure pump gradually, the variation trend of ICP and CPP was recorded.

### 2.11. In Vivo Experimental Design

*Experiment 1 (Figure 1)*: the animals were randomly assigned into five groups: the sham group, rapid decompression group (RDC), controlled decompression for 10 min group (CDC 10 min), controlled decompression for 20 min group (CDC 20 min), and controlled decompression for 30 min group (CDC 30 min). The surgery of the sham group merely included skin incision, skull exposure, bone hole drilling, and the insertion of the ICP probe and balloon for 30 min without inflation. For the other four groups, we inflated the balloon with a pump until the value of ICP reached to the level of 30 mmHg which was maintained for 30 min. As for the RDC group, normal saline (NS) was pumped out rapidly within 3 seconds to reduce ICP suddenly after 30 min balloon-inflation. On the contrary, the ICP was lowered gradually in the CDC group, and the whole process lasted for 10 min, 20 min, or 30 min.

**Figure 2:**
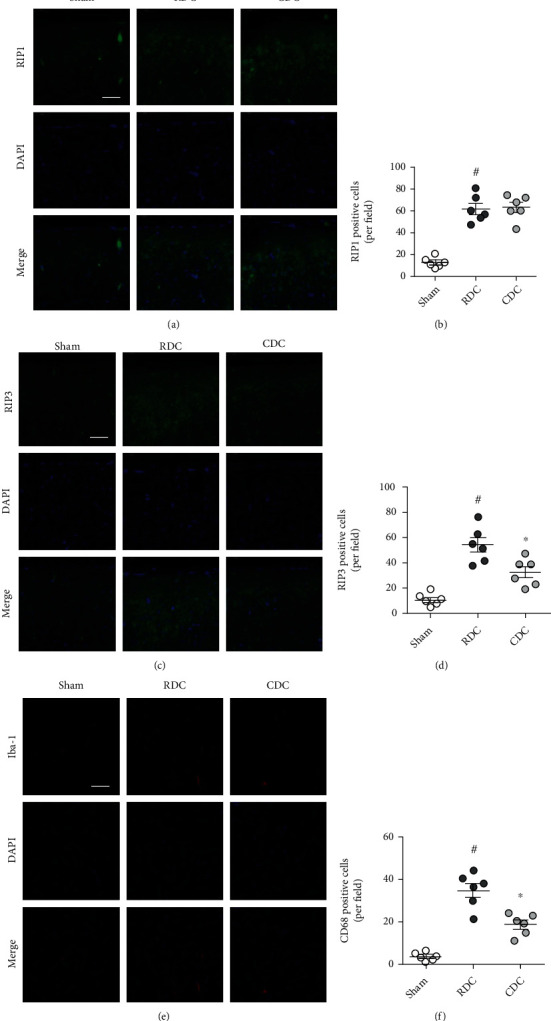
CDC alleviates neuronal necroptosis and neuroinflammation. (a, b) Typical pictures of RIP1 staining (a) and quantification (b) show that CDC had no effect on the RDC-induced increase of RIP1 expression. The scale bar is 50 *μ*m. (c, d) Typical pictures of RIP3 staining (c) and quantification (d) show that CDC attenuates the RIP3 expression compared to RDC. The scale bar is 50 *μ*m. (e, f) Typical pictures of CD68 staining (e) and quantification (f) show that CDC attenuates microglial activation compared to RDC. The scale bar is 50 *μ*m. The data was represented as means ± SEM. ^#^*p* < 0.05 vs. sham group and ^∗^*p* < 0.05 vs. RDC group.

**Figure 3:**
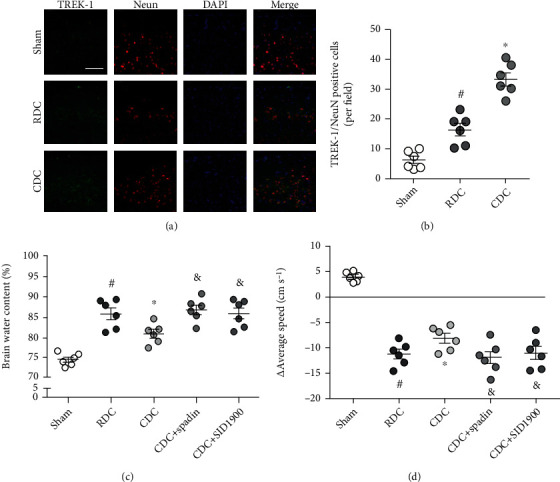
CDC exerts neuroprotective effects via activating TREK-1 in vivo. (a, b) Typical pictures of TREK-1 staining (a) and quantification (b) show that CDC further increased the expression of TREK-1 in cortical neurons compared to RDC. The scale bar is 50 *μ*m. (c) Brain water content assay shows that the CDC-induced inhibition of brain edema was prevented by spadin and SID1900. (d) The neurological assay shows that the CDC-induced preservation of locomotor function was reversed by spadin and SID1900. The data was represented as means ± SEM. ^#^*p* < 0.05 vs. sham group, ^∗^*p* < 0.05 vs. RDC group, and ^&^*p* < 0.05 vs. CDC group.

*Experiment 2 (Figures 2 and 3(a) and 3(b))*: the animals were randomly assigned into three groups: Sham group, RDC group and CDC group. The rats in Sham and RDC group were treated as mentioned in experiment 1. The animals in CDC group were treated with CDC procedure for 30 min as mentioned in experiment 1.

*Experiment 3 (Figures 3(c) and 3(d))*: the animals were randomly assigned into five groups: the sham group, RDC group, CDC group, CDC and spadin group (CDC+spadin), and CDC and SID1900 group (CDC+SID1900). The rats in the first three groups were treated as experiment 2. For the other two groups, rats were pretreated with spadin or SID1900 via the left cerebral ventricle (coordinates relative to bregma: 0.1 mm posterior, 1 mm lateral, and 2 mm deep) before the surgery.

### 2.12. Measurement of Brain Edema

Brian edema was determined by measuring brain water content using the standard wet and dry method [18].

### 2.13. Neurological Function Assay

The CatWalk XT automated gait analysis system (Noldus Information Technology, Wageningen, the Netherlands) was used to measure the locomotor function as previously described [19].

### 2.14. Immunostaining in Brain Sections

The sections were washed three times with PBST for 5 min and were blocked by 10% normal bovine serum (Gibco, United States) in PBST for 1 h at room temperature. After that, the sections were incubated at 4°C overnight in 5% normal goat serum (NGS) in PBST with the following primary antibodies: NeuN (1 : 200, #24307, Cell Signaling), RIP1 (1 : 100, #3493, Cell Signaling), RIP3 (1 : 200, ab62344, Abcam), CD68 (1 : 200, ab125212, Abcam), and TREK-1 (1 : 50, sc-398449, Santa Cruz). After being washed for three times by PBST, the sections were incubated with secondary antibodies conjugated to Alexa Fluor (1 : 800, Invitrogen, United States) for 1 h at 37°C. DAPI was administrated to counterstain the nuclei, and the images were obtained using a Leica SP5 II confocal microscope.

### 2.15. Statistical Analysis

Data represent the mean and standard error of the mean (SEM). Student's *t* test and repeated measure analysis of variance with Student-Newman-Keuls post hoc test were performed for all statistical significance analyses using GraphPad Prism 6.0 software. All experiments were repeated at least for three times.

## 3. Results

### 3.1. Compression Aggravates Neuronal Injury after TNI in Cortical Neurons

**Figure 4:**
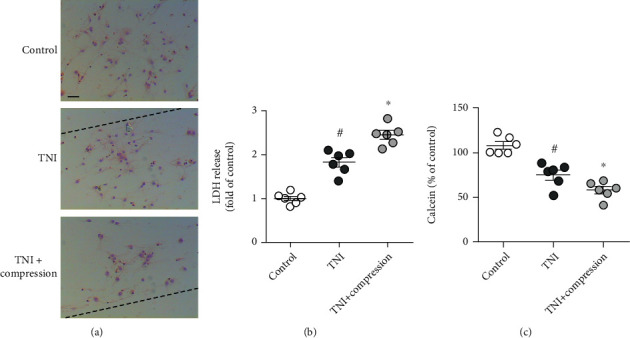
Compression aggravates neuronal injury after TNI in cortical neurons. (a) Typical pictures of H&E staining show that compression aggravates neuronal loss after TNI. The dotted line indicates the edge of the scratch injury. The scale bar is 50 *μ*m. (b) Compression increases LDH release after TNI. (c) Compression decreases calcein signal after TNI. The data was represented as means ± SEM. ^#^*p* < 0.05 vs. control group and ^∗^*p* < 0.05 vs. TNI group.

To mimic intracranial hypertension after TBI in vitro, primary cultured cortical neurons were treated with traumatic injury and compression of 0.5 MPa pressure for 3 h. The results of H&E staining showed that the neuronal loss induced by TNI was significantly increased by compression (Figure 4(a)). LDH release was measured to determine cytotoxicity, and the TNI-induced increase in LDH release in cortical neurons was enhanced by compression (Figure 4(b)). In addition, the neuronal viability was assayed by measuring the calcein AM signaling (Figure 4(c)). The results showed that TNI markedly decreased the calcein signal in cortical neurons, which was further aggravated by compression.

### 3.2. CDC Attenuates Compressive Injury following TNI

**Figure 5:**
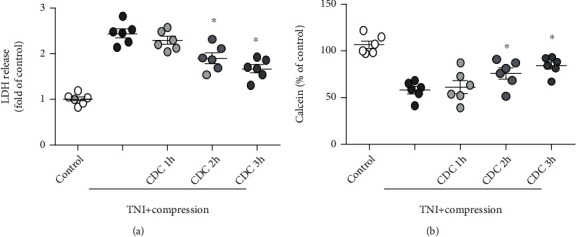
CDC attenuates compressive injury following TNI. (a) CDC for 2 h or 3 h, but not 1 h, attenuates compression-induced increase in LDH release following TNI. (b) CDC for 2 h or 3 h, but not 1 h, attenuates compression-induced decrease in calcein signal following TNI. The data was represented as means ± SEM. ^∗^*p* < 0.05 vs. TNI+compression group.

To mimic controlled decompression in vitro, the 0.5 MPa pressure was gradually released within 1 h (CDC 1 h group), 2 h (CDC 2 h group), or 3 h (CDC 3 h group), while the pressure was completely released at once as control. The results showed that CDC 2 h and CDC 3 h significantly decreased the LDH release induced by TNI and compression, whereas CDC 1 h had no effect (Figure 5(a)). In congruent, CDC 2 h and CDC 3 h, but not CDC 1 h, obviously increased the calcein signal after TNI and compression (Figure 5(b)). CDC 2 h was used in following experiments (represented as CDC).

### 3.3. CDC Alleviates Neuronal Necroptosis

**Figure 6:**
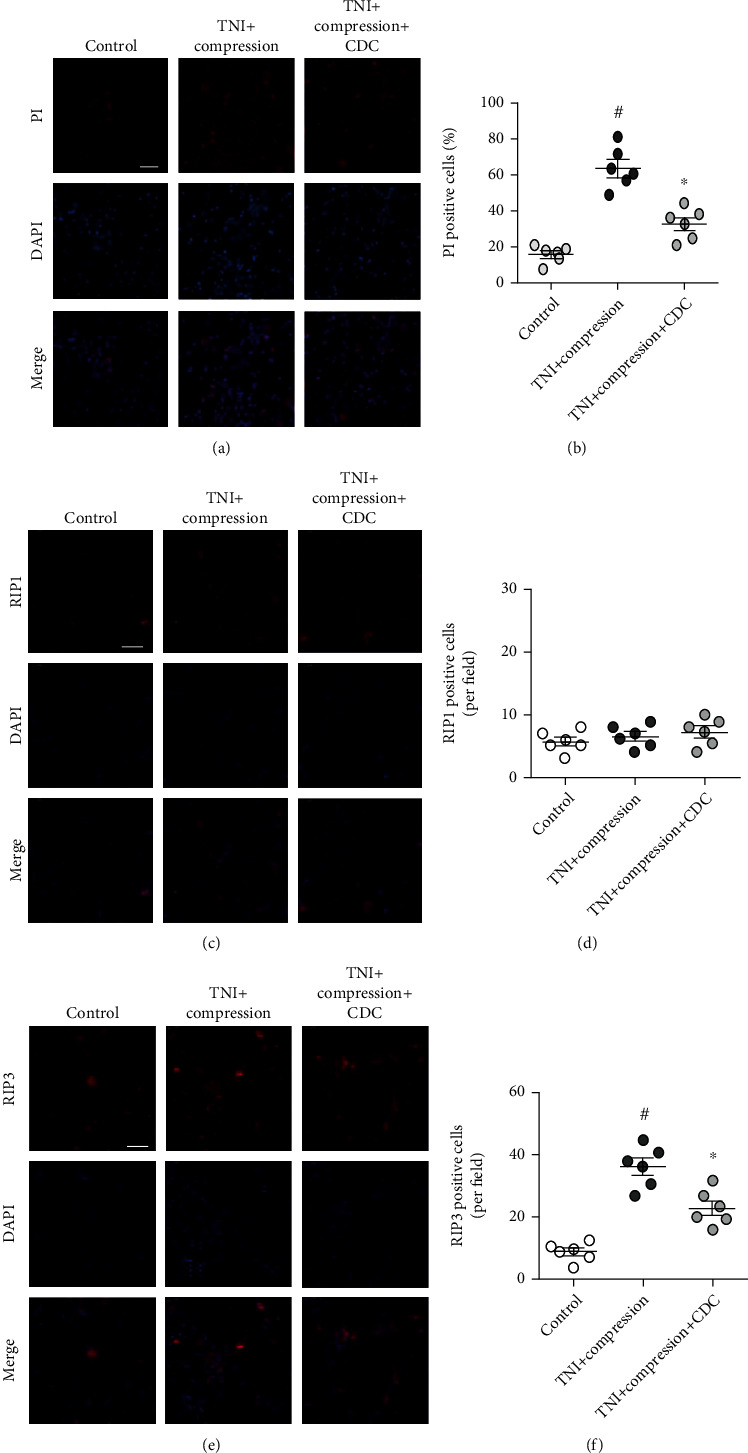
CDC alleviates neuronal necroptosis. (a, b) Typical pictures of PI staining (a) and quantification (b) show that CDC inhibits neuronal necrosis following TNI and compression. The scale bar is 50 *μ*m. (c, d) Typical pictures of RIP1 staining (c) and quantification (d) show that CDC or compressive injury after TNI has no effect on RIP1 expression. The scale bar is 50 *μ*m. (e, f) Typical pictures of RIP3 staining (e) and quantification (f) show that CDC attenuates RIP3 activation after TNI and compression. The scale bar is 50 *μ*m. The data was represented as means ± SEM. ^#^*p* < 0.05 vs. control group and ^∗^*p* < 0.05 vs. TNI+compression group.

Double staining with PI and DAPI was performed to detect necrotic cell death in cortical neurons (Figure 6(a)), and the results showed that the number of PI-positive cells was increased by TNI and compression but significantly decreased by CDC (Figure 6(b)). To investigate the role of necroptosis in our in vitro experiments, we detected the expression of RIP1 (Figure 6(c)) and RIP3 (Figure 6(e)) using immunostaining. The results showed that neither TNI+compression nor CDC had effects on the number of RIP1-positive cells (Figure 6(d)). However, the increased number of RIP3-positive cells was reduced by CDC (Figure 6(f)).

### 3.4. CDC Decreases the Levels of Inflammatory Cytokines

**Figure 7:**
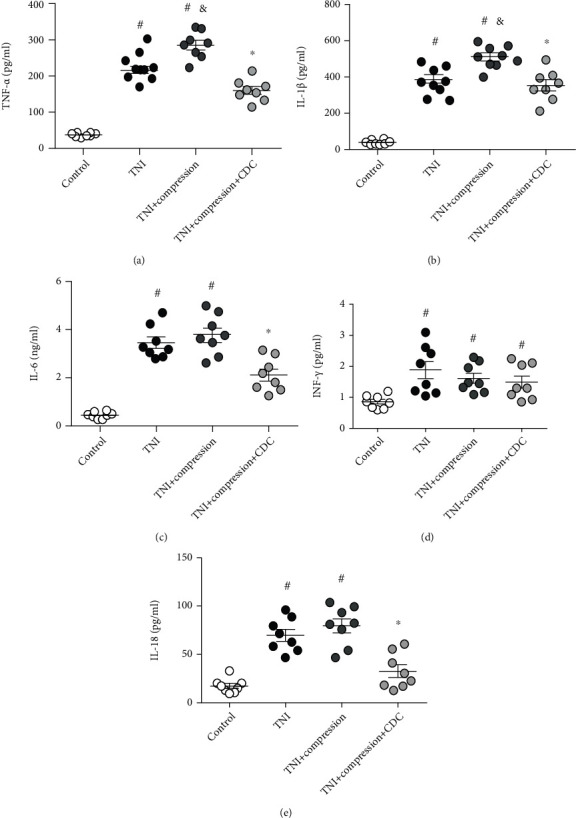
CDC decreases the levels of inflammatory cytokines. (a–c) ELISA assay shows that CDC decreases the levels of TNF-*α* (a), IL-1*β* (b), and IL-6 (c) after TNI and compression. (d) CDC has no effect on INF-*γ* level in cortical neurons after TNI and compression treatment. (e) The TNI and compression-induced increase in IL-18 levels is attenuated by CDC. The data was represented as means ± SEM. ^#^*p* < 0.05 vs. control group, ^&^*p* < 0.05 vs. TNI group, and ^∗^*p* < 0.05 vs. TNI+compression group.

Necroptotic cell death was associated with inflammatory responses. Thus, we measured the levels of inflammatory cytokines in cortical neurons treated with TNI, compression, and/or CDC. The results showed that TNI increased the levels of TNF-*α* (Figure 7(a)) and IL-1*β* (Figure 7(b)), which were further increased by compression. However, these increases were significantly attenuated by CDC. The level of IL-6 was increased by TNI and compression, which was decreased by CDC (Figure 7(c)). In contrast, the increased level of INF-*γ* induced by TNI and compression was not altered by CDC (Figure 7(d)). As shown in Figure 7(e), the increased level of IL-18 after TNI and compression was markedly attenuated by CDC.

### 3.5. CDC Preserves Intracellular Ca^2+^ Homeostasis

**Figure 8:**
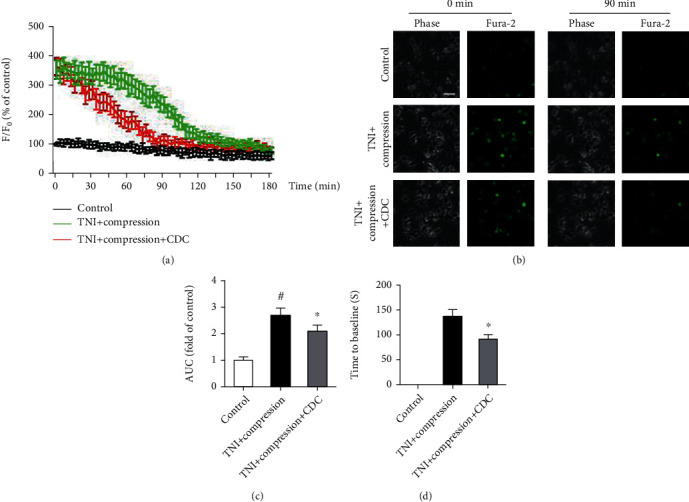
CDC preserves intracellular Ca^2+^ homeostasis. (a) Ca^2+^ imaging shows the intracellular Ca^2+^ levels up to 180 min after TNI and compression. (b) Typical pictures of Ca^2+^ imaging at 0 and 90 min following TNI and compression. The scale bar is 20 *μ*m. (c) CDC attenuates intracellular Ca^2+^ overload up to 180 min following TNI and compression. (d) CDC reduces the time of Ca^2+^ levels to the baseline. The data was represented as means ± SEM. ^#^*p* < 0.05 vs. control group and ^∗^*p* < 0.05 vs. TNI+compression group.

Next, we performed Ca^2+^ imaging to examine the effect of TNI, compression, and CDC on calcium homeostasis (Figure 8(a)). The representative pictures of Ca^2+^ imaging are shown in Figure 8(b). The results showed that TNI and compression-induced Ca^2+^ overload were reduced by CDC (Figure 8(c)). The time of Ca^2+^ signaling to baseline in the CDC group was shorter than that in the TNI and compression group (Figure 8(d)).

### 3.6. CDC Activates TREK-1 Channels

**Figure 9:**
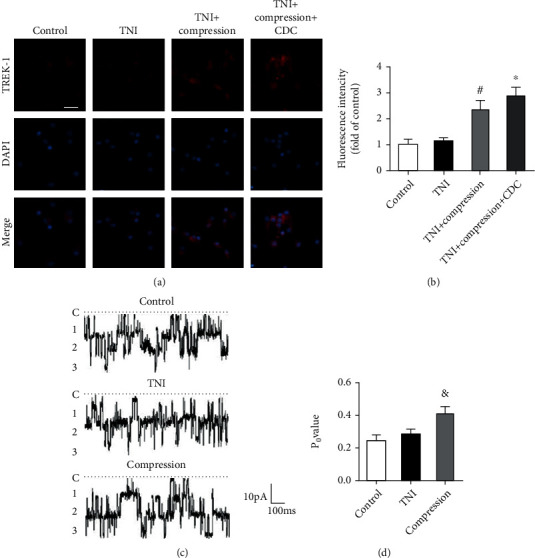
CDC activates TREK-1 channels. (a, b) Typical pictures of TREK-1 staining (a) and quantification (b) show that CDC further enhances the activation of TREK-1 channels following TNI and compression. The scale bar is 20 *μ*m. (c, d) Typical recordings from the inside-out patch illustrating TREK-1 channel activation (c) and quantification (d) show that compression, but not TNI, activates TREK-1 channels in cortical neurons. The data was represented as means ± SEM. ^#^*p* < 0.05 vs. TNI group, ^∗^*p* < 0.05 vs. TNI+compression group, and ^&^*p* < 0.05 vs. control group.

To detect the potential involvement of TREK-1 in our findings, we performed immunostaining using the TREK-1 antibody (Figure 9(a)). The results showed that TNI did not change the fluorescence intensity of TREK-1 in cortical neurons, whereas compression significantly increased the TREK-1 signal after TNI (Figure 9(b)). The compression-induced increase in TREK-1 fluorescence intensity after TNI was further enhanced by CDC. In addition, we investigated whether TNI or compression has effects on TREK-1 channel activity, which was detected by inside-out membrane patches at a physiological steady membrane voltage of -40 mV (Figure 9(c)). The results showed that the TREK-1 channel activation was markedly increased by compression, but not by TNI (Figure 9(d)).

### 3.7. CDC Inhibits Ca^2+^ Responses via TREK-1 in Cortical Neurons

**Figure 10:**
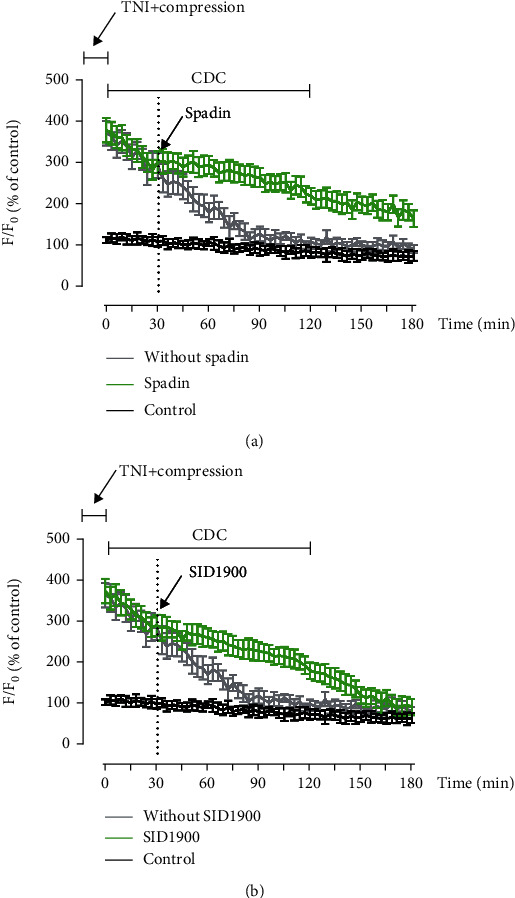
CDC inhibits Ca^2+^ responses via TREK-1. (a) Ca^2+^ imaging shows that CDC-induced attenuation of intracellular Ca^2+^ overload was ablated by the TREK-1 blocker spadin. (b) Ca^2+^ imaging shows that CDC-induced attenuation of intracellular Ca^2+^ overload was ablated by the TREK-1 blocker SID1900. The data was represented as means ± SEM.

To investigate the role of the TREK-1 channel in CDC-induced regulation of intracellular Ca^2+^ homeostasis after TNI, we repeated the Ca^2+^ imaging experiments using the TREK-1 blockers, spadin and SID1900. The results showed that the decreased intracellular Ca^2+^ concentration induced by compression after TNI was significantly preserved by both spadin (1 *μ*M, Figure 10(a)) and SID1900 (30 *μ*M, Figure 10(b)).

### 3.8. Involvement of TREK-1 in CDC-Induced Protection In Vitro

**Figure 11:**
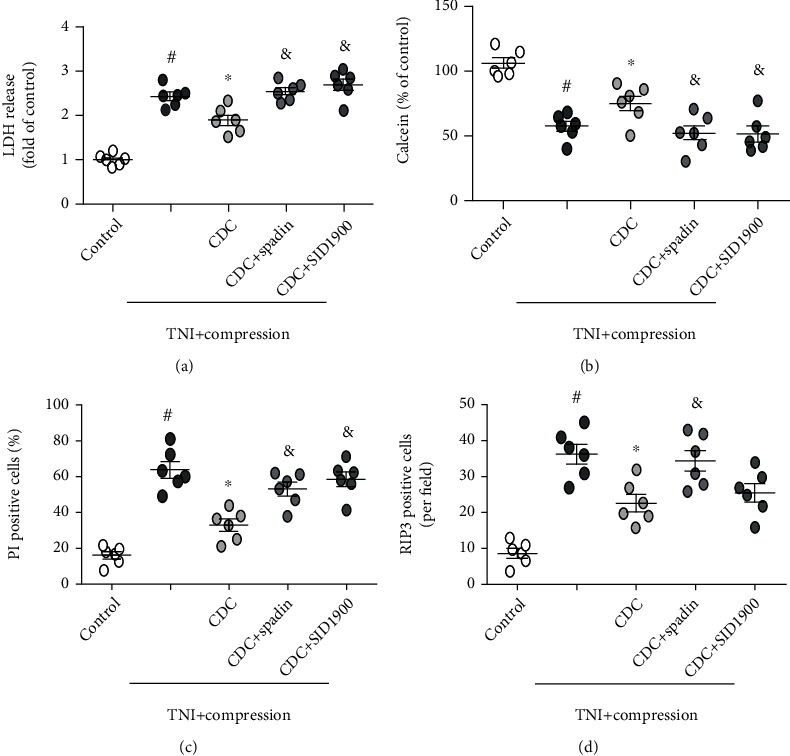
Involvement of TREK-1 in CDC-induced protection. (a) The CDC-induced decrease in LDH release was prevented by spadin and SID1900. (b) The CDC-induced increase in calcein signal was prevented by spadin and SID1900. (c) The CDC-induced inhibition of RIP3 activation was prevented by spadin and SID1900. (d) The CDC-induced inhibition of neuronal necrosis was prevented by spadin, but not by SID1900. The data was represented as means ± SEM. ^#^*p* < 0.05 vs. control group, ^∗^*p* < 0.05 vs. TNI+compression group, and ^&^*p* < 0.05 vs. CDC+TNI+compression group.

Treatment with spadin and SID1900 (at the beginning of CDC) was used to investigate the involvement of TREK-1 in CDC-induced protection and related mechanisms. The results showed that the CDC-induced decrease in cytotoxicity, as evidenced by decreased LDH release, was prevented by spadin and SID1900 (Figure 11(a)). Congruently, the CDC-induced increase in calcein signal (Figure 11(b)), as well as the CDC-induced decrease in the number of PI-positive cells (Figure 11(c)), was apparently reversed by spadin and SID1900. In addition, the CDC-induced inhibition of RIP3 expression was markedly attenuated by spadin, but not by SID1900 (Figure 11(d)).

### 3.9. CDC Attenuates Brain Damage after Traumatic Intracranial Hypertension

To confirm the above findings in in vivo conditions, we established a traumatic intracranial hypertension model in rats, and the horizontal and coronal schematic diagrams are shown in Figure 1(a). The results of the brain water content assay showed that CDC for 20 min or 30 min, but not CDC for 10 min, significantly reduced brain edema compared to the RDC group (Figure 1(b)). The neurological assay showed that CDC for 20 min or 30 min, but not CDC for 10 min, preserved locomotor function compared to RDC (Figure 1(c)). Next, we performed immunostaining using the NeuN antibody to detect the neuronal loss following traumatic intracranial hypertension (Figure 1(d)), and the results showed that CDC for 20 min or 30 min, but not CDC for 10 min, inhibited neuronal loss compared to RDC (Figure 1(e)). The CDC for 30 min was used in the following experiments.

### 3.10. CDC Alleviates Neuronal Necroptosis and Neuroinflammation In Vivo

To investigate the effects of CDC on neuronal necroptosis following traumatic intracranial hypertension, the expression of RIP1 (Figure 2(a)) and RIP3 (Figure 2(c)) on brain sections was detected by immunostaining using corresponding antibodies. The results showed that RDC and CDC both increased the expression of RIP1 compared to the sham group, but there is no difference between these two groups (Figure 2(b)). However, CDC markedly decreased the expression of RIP3 compared to RDC (Figure 2(d)). The activation of microglia was determined by immunostaining using the CD68 antibody (Figure 2(e)), and the results showed that microglial activation in the CDC group was lower than that in the RDC group (Figure 2(f)).

### 3.11. CDC Exerts Neuroprotective Effects via Activating TREK-1 In Vivo

We also performed immunostaining using the TREK-1 antibody in brain sections after traumatic intracranial hypertension (Figure 3(a)), and the results showed that RDC and CDC both increased the expression of TREK-1 with the higher levels of TREK-1 in the CDC group (Figure 4(b)). It was shown that the increased expression of TREK-1 was mainly in neurons, as evidenced by colocalization with NeuN staining (Figure 3(a)). To further confirm the involvement of TREK-1 in vivo, we repeated the brain water content assay (Figure 3(c)) and neurological function assay (Figure 3(d)) using spadin and SID9100. The result showed that the effects of CDC on brain edema and locomotor impairment were weakened by spadin and SID9100.

## 4. Discussion

Uncontrollable intracranial hypertension is one of the most important causes of death in TBI patients, but rapid decompression during the standard decompressive craniectomy surgery has been reported to be associated with many postoperative complications [20, 21]. In this study, we demonstrated that compression aggravates neuronal injury after TNI in cortical neurons and identified CDC as an effective strategy to prevent this kind of neuronal injury. We found that (a) CDC (2 h and 3 h) effectively reduces compressive neuronal damage following TNI, (b) CDC significantly inhibits neuronal necrosis and RIP3 activation;,(c) CDC markedly decreases the levels of inflammatory cytokines, (d) compression-induced intracellular Ca^2+^ overload is attenuated by CDC in vitro, (e) CDC enhances the expression and activity of TREK-1 channel induced by compression, (f) the CDC-induced effects on Ca^2+^ metabolism and neuronal injury following TNI and compression are decreased by TREK-1 blockers, (g) CDC (20 min and 30 min) is effective in alleviating brain edema and locomotor impairment in vivo, (h) CDC inhibits neuronal necroptosis and microglial activation and increases TREK-1 expression, and (i) the CDC-induced protection in vivo is reversed by TREK-1 blockers.

At the beginning of the 19th century, decompressive craniectomy was firstly described by Kocher T and Harvey Cushing to treat patients with low Glasgow coma scale (GCS) score and dilated pupils following TBI. However, the standard procedure of rapid decompression was found to be associated with many intraoperative and postoperative complications, including delayed intracranial hematoma and diffuse brain swelling. In the past few decades, neurosurgeons have made a lot of improvements to the surgical procedures. Alves et al. used “Basal durotomy”, a novel design of dural opening with a “reversed U-shaped” durotomy incision, to minimize the risk of massive intraoperative swelling [22]. In 2013, decompressive craniectomy with multi-dural stabs (also called SKIMS technique) was introduced to be effective in increasing survival of low GCS score and severe TBI patients with acute subdural hematoma [23]. Our previous studies showed that decompressive craniectomy using the CDC strategy, a method to gradually reduce the intracranial pressure (ICP) with the ventricular or brain tissue ICP detector, significantly improved outcomes and reduced complications in severe TBI patients [5]. Here, we used the in vitro model to confirmed the effectiveness of CDC in TNI- and compression-treated cortical neurons. Our results showed that CDC for 2 and 3 h markedly reduced LDH release and increased calcein signal, which were accompanied by inhibited neuronal death. In addition, these protective effects were also confirmed in an in vivo traumatic intracranial hypertension model in rats with the CDC procedure for 20 min or 30 min. Thus, CDC could be an effective strategy for the treatment of compressive neuronal injury following TBI both in vitro and in vivo.

Regulated neuronal death is thought to be an ideal therapeutic target for neurological disorders. However, the primary brain damage following TBI is mainly due to neuronal necrosis, an uncontrollable cell death characterized by ATP depletion, intracellular organelle swelling, and loss of membrane integrity [24–26]. Necroptosis is the first form of programmed necrosis with an important role in both physiological and pathological conditions [27]. More recently, the necroptosis inhibitor necrostatin-1 was predicted to have the potential to protect against the complications of coronavirus disease 2019 (COVID-19) [28]. In addition, necroptosis has been demonstrated to contribute to the delayed neuronal death in multiple neurological disorders, ranging from chronic neurodegenerative disorders such as Alzheimer's disease (AD) and Parkinson's disease (PD) to acute insults, including stroke and TBI [29, 30]. The necroptosis inhibitors were found to exert neuroprotective effects against TBI [31], and blocking necroptotic neuronal death was shown to mediate the therapeutic potential of many treatments for TBI, such as hypothermia [32]. In this study, increased number of PI-positive cells was found in TNI- and compression-treated neurons, confirming the involvement of necroptosis in our in vitro traumatic intracranial hypertension conditions. The necroptotic pathway depends on the assembly of the apical protein kinases, receptor-interacting protein kinase 1 (RIP1) and RIP3, to form the high molecular weight complex necrosome, which in turn governs the oligomerization and translocation of the cell death executor mixed lineage kinase domain-like (MLKL) [33]. Intriguingly, our results showed that TNI and compression increased RIP3 (but not RIP1) activation, which was partially prevented by CDC. In addition, increased expression of RIP1 and RIP3 was found after traumatic intracranial hypertension in vivo, while CDC only decreased RIP3 expression. These data indicated that CDC-induced inhibition of neuronal necroptosis might be mediated by a RIP1-independent mechanism, which was also previously reported [34, 35].

As the stretch-dependent K^+^ channels (with TREK-2 and TRAAK), TREK-1 was originally described in rat cardiac ventricular muscle, where mechanical stretch could increase its expression in mechanoelectrical coupling [36]. Following researches demonstrated that the mechanogating activity of TREK-1 channels was also important for the sensation of pain in sensory neurons [37]. Thus, to determine the potential involvement of TREK-1, we detected the expression and activity of TREK-1. We found that TNI had no effect on the expression and activity of TREK-1, but compression significantly potentiated TREK-1 activation, which was prolonged by CDC strategy. Immunostaining results in brain sections also showed that CDC markedly increased the expression of TREK-1 in neurons. The TREK-1-induced K^+^ efflux could counter-balance the detrimental arrhythmogenic cation influx through non-selective cation channels, such as Na^+^ channels [38]. In addition, TREK-1 was demonstrated to mediate the fast glutamate release in astrocytes via the activation of G-protein-coupled receptors (GPCRs) [39]. These data suggest that TREK-1 might be involved in glutamate-associated neurotoxicity. To confirm this hypothesis, we used two TREK-1 specific inhibitors, spadin and SID1900. Spadin is a synthetic peptide derived from sortilin which was shown to inhibit TREK-1 with high affinity [11]. The small molecule SID1900 was found to inhibit TREK-1 with an IC_50_ of 29.72 *μ*M, comparing that of spadin is 40 nM [40]. The results showed that CDC-induced effects on Ca^2+^ regulation, neuronal injury, as well as the in vivo protective effects, were all partially reversed by spadin and SID1900, confirming the role of TREK-1 in CDC-induced neuroprotection. Intriguingly, a previous study has reported the negative results of TBI-related brain damage in mice lacking TREK-1 [41]. However, deficiency of TREK-1 was shown to exacerbate BBB injury and neuroinflammation in intracranial hemorrhage [42]. The role of TREK-1 in neuronal injury seems to be controversial in different experimental models, where mechanical force could be an important factor.

As the most abundant divalent cation in mammalian cells, Ca^2+^ serves as the key intracellular signaling molecule with a striking 10000-fold concentration gradient larger than other ions, including Na^+^ and K^+^ [43]. The physiological function of Ca^2+^ in the central nervous system depends on where, when, and how it enters and exits the neurons and glial cells. Increasing evidence indicate that dysfunction of intracellular Ca^2+^ homeostasis is involved in TBI-induced brain damage, and many Ca^2+^ channel blockers are thought to exert neuroprotective potential [44]. Our previous study showed that compression resulted in intracellular Ca^2+^ overload via promoting intracellular Ca^2+^ release in primary cultured cortical neurons [16]. Here, we detected the changes in intracellular Ca^2+^ concentrations using Ca^2+^ imaging, and the results showed that compressive injury following TNI caused an increase in the *F*/*F*_0_ signal, which was attenuated by CDC. A previous study showed that TREK-1 gene knockout impairs neuronal excitability, synaptic plasticity, and cognitive function [12]. The activity of TREK-1 channels can be regulated by various chemical stimuli, including Gq-coupled group I mGluRs, mGluR1, and mGluR5, which enhance the release of Ca^2+^ from the intracellular calcium stores by stimulating 1,4,5-trisphosphate receptors (IP_3_R) on the endoplasmic reticulum (ER) membrane [45]. Thus, we repeated the Ca^2+^ imaging experiments using TREK-1 inhibitors, and the intracellular Ca^2+^ overload was found to be prolonged after blocking TREK-1 activation. Congruently, TREK-1 was shown to regulate pressure sensitivity and Ca^2+^ signaling in trabecular meshwork cells [46], and the TREK-1-specific blocker spadin was found to potentiate Ca^2+^ influx and insulin secretion in pancreatic beta cells [47]. These data strongly suggest that the TREK-1-mediated neuroprotective mechanism was associated with the preservation of intracellular Ca^2+^ homeostasis, possibly via regulating Ca^2+^ release form intracellular Ca^2+^ stores, which needs to be further determined.

## 5. Conclusions

In summary, our present data indicate that CDC for 2 h or 3 h in vitro and CDC for 20 min or 30 min in vivo exert neuroprotective effects. The potential underlying mechanisms involve in TREK-1 mediated regulation of intracellular Ca^2+^ homeostasis and inhibition of neuronal necroptosis.

## Data Availability

The research data used to support the findings of this study are included within the article.
